# Change in longitudinal trends in sleep quality and duration following breast cancer diagnosis: results from the Women’s Health Initiative

**DOI:** 10.1038/s41523-018-0065-7

**Published:** 2018-06-29

**Authors:** Chloe M. Beverly, Michelle J. Naughton, Michael L. Pennell, Randi E. Foraker, Gregory Young, Lauren Hale, Elizabeth M. Cespedes Feliciano, Kathy Pan, Tracy E. Crane, Suzanne C. Danhauer, Electra D. Paskett

**Affiliations:** 1 Division of Epidemiology, College of Public Health, The Ohio State University, Columbus, OH 43210 USA; 2 Division of Population Sciences, Department of Internal Medicine, College of Medicine, The Ohio State University, Columbus, OH 43210 USA; 3 Division of Biostatistics, College of Public Health, The Ohio State University, Columbus, OH 43210 USA; 4 Institute for Informatics, School of Medicine, Washington University in St. Louis, St. Louis, MO 63108 USA; 5 Center for Biostatistics, The Ohio State University, Columbus, OH 43210 USA; 6 Program in Public Health, Department of Family, Population, and Preventive Medicine, Stony Brook University, Stony Brook, NY 11794 USA; 7 Kaiser Permanente Northern California Division of Research, Oakland, CA 94612 USA; 8 Los Angeles Biomedical Research Institute at Harbor-UCLA Medical Center, University of California, Los Angeles, Torrance, CA 90509 USA; 9 College of Nursing, University of Arizona Cancer Center, University of Arizona, Tucson, AZ 85724 USA; 10 Department of Social Sciences and Health Policy, Division of Public Health Sciences, Wake Forest School of Medicine, Winston-Salem, NC 27157 USA; 11 Division of Cancer Prevention and Control, Department of Internal Medicine, College of Medicine, The Ohio State University, Columbus, OH 43210 USA

## Abstract

Breast cancer survivors frequently report sleep problems, but little research has studied sleep patterns longitudinally. We examined trends in sleep quality and duration up to 15 years before and 20 years after a diagnosis of breast cancer, over time among postmenopausal women participating in the Women’s Health Initiative (WHI). We included 12,098 participants who developed invasive breast cancer after study enrollment. A linear mixed-effects model was used to determine whether the time trend in sleep quality, as measured by the WHI Insomnia Rating Scale (WHIIRS), a measure of perceived insomnia symptoms from the past 4 weeks, changed following a cancer diagnosis. To examine sleep duration, we fit a logistic regression model with random effects for both short (<6 h) and long (≥9 h) sleep. In addition, we studied the association between depressive symptoms and changes in WHIIRS and sleep duration. There was a significantly slower increase in the trend of WHIIRS after diagnosis (*β* = 0.06; *p* = 0.03), but there were non-significant increases in the trend of the probability of short or long sleep after diagnosis. The probability of depressive symptoms significantly decreased, though the decrease was more pronounced after diagnosis (*p* < 0.01). Trends in WHIIRS worsened at a relatively slower rate following diagnosis and lower depression rates may explain the slower worsening in WHIIRS. Our findings suggest that over a long period of time, breast cancer diagnosis does not adversely affect sleep quality and duration in postmenopausal women compared to sleep pre-diagnosis, yet both sleep quality and duration continue to worsen over time.

## Epidemiology: Long-term sleep patterns unaffected by breast cancer diagnosis

Despite frequent reports of poor sleep among survivors of breast cancer, a large epidemiological study has found no evidence that diagnosis of invasive breast cancer long-term sleep problems. Chloe Beverly of The Ohio State University in Columbus, USA, and colleagues examined long-term patterns in sleep quality, sleep duration and symptoms of depression among more than 12,000 participants of the Women’s Health Initiative who were diagnosed with breast cancer. On the whole, they found that women developed insomnia at a slightly slower rate after their diagnosis and the prevalence of depression went down, with little change in sleep duration. Although some women may experience cancer-related sleep disturbances shortly after their diagnosis and treatment, the findings suggest that, over the long term, most sleep issues remain consistent compared to before diagnosis.

## Introduction

Breast cancer is the most common cancer diagnosis in women.^1^ Breast cancer survivors report sleep problems, including poor sleep quality, poor sleep efficiency, and sleep disturbance,^2^ as one of their top five most burdensome long-term health issues.^3^ Compared to women without cancer, breast cancer survivors are twice as likely to experience sleep problems, and prior studies demonstrated that 20–90% report sleep disturbances, depending on the study assessment method.^3–6^ Sleep problems may contribute to poorer quality of life and health status among survivors.

Sleep changes in women with breast cancer can begin shortly after diagnosis^7^ or during initial treatment with chemotherapy or radiation therapy and may become chronic.^8^ Chemotherapy has been shown to disrupt circadian rhythms^9^ and increase menopausal symptoms, which in turn impact sleep quality and quantity.^10^ In one of the few longitudinal studies on this topic, researchers found that some sleep problems, such as inadequate duration and lack of restorative sleep, resolve soon after the short-term stressors of a cancer diagnosis and active treatment are completed, but other sleep problems that originally stem from cancer-related issues (such as treatments) persist.^11^ Little is known about the longer term health effects of sleep disturbance on breast cancer survivors.^12^

Currently, there are limited longitudinal data on sleep problems of breast cancer survivors in older adults by various ages. Well-documented changes in sleep structure, sleep quality, and sleep timing are associated with increasing age^13^ and the highest rates of insomnia are in postmenopausal women.^14,15^ Among older women in good health with no insomnia complaints, there are more awakenings during sleep than among younger adults.^16^ In general, individuals can expect more disturbed sleep with age, including frequent awakenings, early morning awakenings, and increased daytime napping. However, individuals with comorbid conditions have more exacerbated sleep changes than those in good health.^17^

Three papers have been published on breast cancer and sleep using data from the Women’s Health Initiative (WHI), a national longitudinal cohort study of postmenopausal women.^18–20^ Researchers found no association between self-reported sleep duration, sleep quality, insomnia, or sleep disturbance and risk of breast cancer.^18^ In relation to cancer survival, short sleep combined with frequent snoring was negatively associated with breast cancer survival,^19^ and more aggressive breast cancer tumors were seen in women with shorter sleep duration and poorer quality sleep.^20^ These prior research studies, however, do not identify how sleep patterns may change over long periods of time following a breast cancer diagnosis. The present study uses WHI longitudinal data to examine sleep measures spanning up to 15 years before and 20 years after a breast cancer diagnosis. The objective of this study was to determine whether trends in sleep quality and duration changed after cancer diagnosis among 12,098 women in the WHI diagnosed with invasive breast cancer after enrollment. Since the relationship between depression and sleep has been well-established,^21–23^ we also considered this important to explore.

## Results

### Demographics

Baseline characteristics of the study population are listed in Table 1. A majority of women were non-Hispanic White (87.4%), enrolled into WHI at ages 60–69 (46.7%), and were part of the WHI observational study (OS) (58.3%). The mean age at breast cancer diagnosis was 70.3 years and localized breast cancer was the most common stage at diagnosis (74.8%). About 10% of women had Burnam scores above the cutoff for depressive symptomology (≥0.06). The average number of sleep measures per woman was 2.9 (range: 1–8). At WHI baseline, women had an average WHIIRS sleep quality score of 6.5 and about 30% of women self-reported insomnia symptoms. There were 18.4% of women in the highest insomnia severity category. Most women slept between 7 and 8 h per night (62.6%). The prevalence of short (<6 h) and long (≥9 h) sleep duration was 7.0 and 4.4%, respectively.

**Table 1:** Baseline demographic, clinical, and sleep variables of study population

	All participants *n* = 12,098^a^
*Demographic factors*	
*Race, n (%)*	
American Indian/Alaskan Native	37 (0.3)
Asian/Pacific Islander	256 (2.1)
Black or African American	841 (7.0)
Hispanic/Latina	282 (2.3)
Non-Hispanic white	10,555 (87.4)
Other	104 (0.9)
*Age at enrollment (years), n (%)*	
50–54	1548 (12.8)
55–59	2529 (20.9)
60–69	5651 (46.7)
70–79	2370 (19.6)
*Trial arm, n (%)*	
Clinical trial	5051 (41.7)
Observational study	7047 (58.3)
*Clinical factors*	
Age at BC diagnosis (years), mean (SD)	70.3 (7.8)
Years in WHI until BC diagnosis, mean (SD)	7.9 (5.0)
First sleep measure following BC diagnosis (in years), mean (SD)	5.8 (4.4)
*Stage of cancer, n (%)*	
Localized	7199 (74.8)
Regional	2180 (22.7)
Distant	141 (1.5)
Unknown	106 (1.1)
*Medications for sleep, n (%)*	
Not in past 4 week	9212 (76.1)
Less than once per week	1149 (9.5)
1–2 times per week	674 (5.6)
3–4 times per week	279 (2.3)
5+ times per week	784 (6.5)
Depressive symptomology, *n* (%) (Burnam scale cutoff, 0.06)	1150 (9.7)
*Sleep factors*	
WHIIRS score, mean (SD)	6.5 (4.4)
Insomnia (≥9), *n* (%)	3572 (29.5)
Insomnia severity, *n* (%)	
0 (0–3)	3383 (28.0)
1 (4–6)	3383 (28.0)
2 (7–10)	3110 (25.7)
3 (**≥**11)	2222 (18.4)
*Sleep duration, n (%)*	
≤5 h	849 (7.0)
6 h	3149 (26.0)
7 h	4737 (39.2)
8 h	2832 (23.4)
≥9 h	531 (4.4)

^a^Not all participants answered every question

### Comparison of sleep patterns by race/ethnicity

Both baseline WHIIRS sleep quality score (*p* = 0.02) and duration (*p* < 0.01) differed by race/ethnicity (Table 2). American Indian/Alaskan Natives and non-Hispanic white women had the highest mean baseline WHIIRS scores at 6.6, while Asian/Pacific Islanders had the lowest mean baseline WHIIRS score of 5.6 points. Black/African Americans had the greatest proportion of women sleeping <6 h per night at baseline (19.6%). American Indian/Alaskan Natives had the lowest proportion of women sleeping between 7 and 8 h (35.1%) and also the highest proportion sleeping ≥9 h (8.1%). Baseline insomnia symptoms did not differ by race/ethnicity (*p* = 0.11).

**Table 2:** Baseline sleep variables of participants by race/ethnicity and age group

	Race/ethnicity^a^							Age at enrollment, years^a^				
	AI/AN (*n* = 37)	A/PI (*n* = 256)	Black/AA (*n* = 841)	Hispanic (*n* = 282)	NHW (*n* = 10,555)	Other (*n* = 104)	*p*-value	50–54 (*n* = 1548)	55–59 (*n* = 2529)	60–69 (*n* = 5651)	70–79 (*n* = 2370)	*p*-value
Sleep factors												
WHIIRS score, mean (SD)	6.6 (6.0)	5.6 (4.1)	6.4 (4.6)	6.5 (4.8)	6.6 (4.3)	6.2 (4.2)	0.02	6.3 (4.5)	6.3 (4.4)	6.5 (4.3)	6.9 (4.4)	<0.01
Insomnia (≥9), *n* (%)	13 (35.1)	57 (22.3)	245 (29.1)	77 (27.3)	3148 (29.8)	27 (26.0)	0.11	424 (27.4)	704 (27.8)	1675 (29.6)	769 (32.5)	<0.01
Insomnia severity, *n* (%)												
0 (0–3)	15 (40.5)	92 (35.9)	246 (29.3)	84 (29.8)	2911 (27.6)	31 (29.8)		486 (31.4)	771 (30.5)	1547 (27.4)	579 (24.4)	<0.01
1 (4–6)	4 (18.8)	74 (28.9)	249 (29.6)	79 (28.0)	2930 (27.8)	34 (32.7)	0.02	416 (26.9)	703 (27.8)	1608 (28.5)	656 (27.7)	
2 (7–10)	10 (27.0)	58 (22.7)	190 (22.6)	65 (23.1)	2765 (26.2)	19 (18.3)		379 (24.5)	601 (23.8)	1447 (25.6)	683 (28.8)	
3 (≥11)	8 (21.6)	32 (12.5)	156 (18.6)	54 (19.2)	1949 (18.5)	20 (19.2)		267 (17.2)	454 (18.0)	1049 (18.6)	452 (19.1)	
Sleep duration, *n* (%)												
≤5 h	5 (13.5)	38 (14.8)	165 (19.6)	32 (11.4)	595 (5.6)	11 (10.6)		114 (7.4)	178 (7.0)	375 (6.6)	182 (7.7)	<0.01
6 h	16 (43.2)	103 (40.2)	311 (37.0)	79 (28.0)	2604 (24.7)	34 (32.7)	<0.01	426 (27.5)	704 (27.8)	1394 (24.7)	625 (26.4)	
7 h	8 (21.6)	86 (33.6)	232 (33.6)	110 (39.0)	4254 (40.3)	35 (33.7)		644 (41.6)	986 (39.0)	2210 (39.1)	897 (37.9)	
8 h	5 (13.5)	23 (9.0)	103 (12.3)	55 (19.5)	2622 (24.8)	18 (17.3)		324 (20.9)	578 (22.9)	1388 (24.6)	542 (22.9)	
≥9 h	3 (8.1)	6 (2.3)	30 (3.6)	6 (2.1)	480 (4.5)	6 (5.8)		40 (2.6)	83 (3.3)	284 (5.1)	124 (5.2)	

*AI/AN* American Indian/Alaskan Native, *A/PI* Asian/Pacific Islander, *Black/AA* Black or African American, *Hispanic* Hispanic/Latina, *NHW* non-Hispanic white, *Other* other race

^a^Not all participants answered every question

### Comparison of sleep patterns by age at enrollment

WHIIRS scores differed by age group (*p* < 0.01), with the highest scores observed among women aged 70–79 years (mean = 6.9) and the lowest among women aged 50–54 years (mean = 6.3, see Table 2). Baseline insomnia symptoms and sleep duration also differed by age group (*p* < 0.01 for each test), with women aged 70–79 years the most likely group to experience insomnia symptoms (32.5%), sleep <6 h per night (7.7%), and to sleep ≥9 h (5.2%). Women aged 60–69 years had the highest proportion of women sleeping between 7 and 8 h (63.7%).

### Change in longitudinal trend in WHIIRS sleep quality

**Fig. 1:**
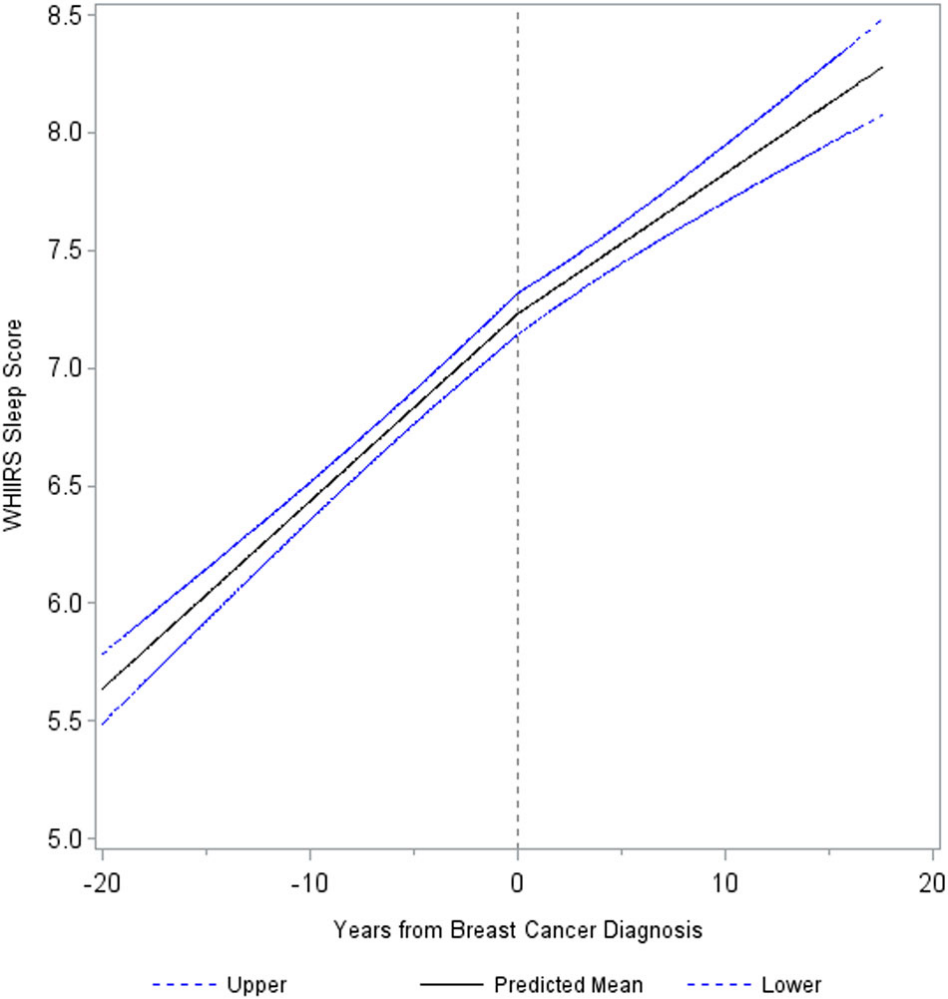
Overall trend in WHIIRS sleep score by years from diagnosis with 95% confidence interval bounds. There was a significant change in trend of WHIIRS score after diagnosis (*p*-interaction = 0.03). Before diagnosis, the score was increasing (*p*-interaction < 0.01) at 0.08 points per year and after diagnosis the score was still increasing (*p*-interaction < 0.01), but at a slower rate of 0.06 points per year. Vertical center line indicates breast cancer diagnosis time point.

Data from these WHI participants were available for up to 15 years before and 20 years after breast cancer diagnosis. The linear mixed model estimated a mean WHIIRS score at breast cancer diagnosis of 7.23 points (95% CI: 7.14–7.32). This is below the 9-point cutoff for insomnia, but still indicates frequent sleep disturbance over the last month. There was a significant change in trend of WHIIRS score after diagnosis (β-interaction = −0.02, 95% CI: −0.04–−0.002, *t*(6182) = −2.17, *p*-interaction = 0.03; see Fig. 1). Before diagnosis, the score was increasing at 0.08 points per year (95% CI: 0.07–0.09, *t*(5490) = 16.88, *p* < 0.01), and after diagnosis the score was still increasing, but at a slower rate of 0.06 points per year (95% CI: 0.05–0.07, *t*(3900) = 9.31, *p* < 0.01). The change in trend of WHIIRS score after breast cancer diagnosis did not differ significantly by race/ethnicity, age at diagnosis, cancer stage at diagnosis, education level, income level, BMI category, marital status, physical activity, and alcohol drinking (Holm’s corrected *p*-values all >0.8).

### Change in longitudinal trend in sleep duration

**Fig. 2:**
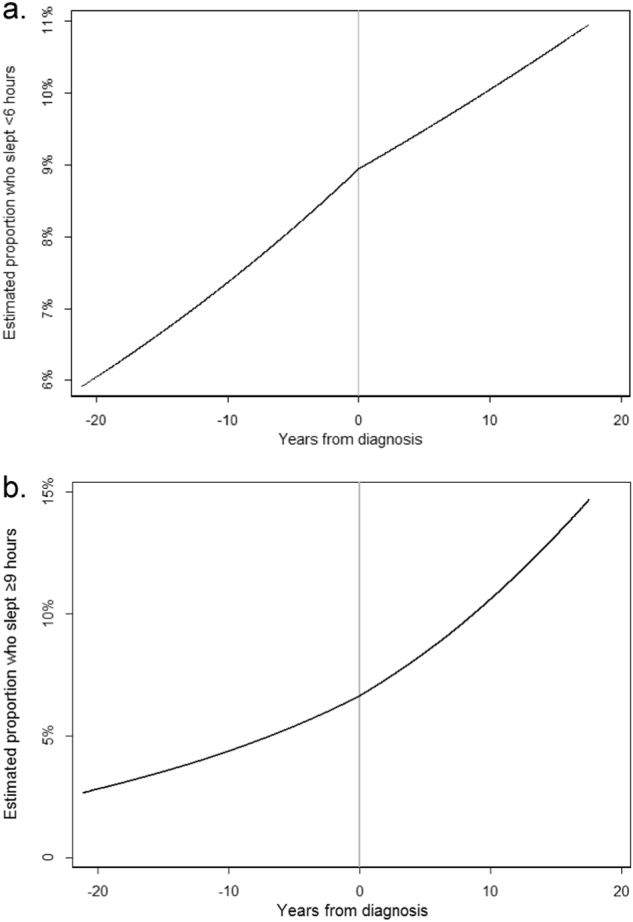
**a** Sleep duration—overall probability of <6 h sleep by years from diagnosis. Prior to diagnosis, the estimated probability of short sleep increased from 6% at 20 years prior to 9% at diagnosis. Following diagnosis, the probability increased at a slower rate, and only increased to 11% about 20 years after diagnosis (*p* = 0.29). Vertical center line indicates breast cancer diagnosis time point. **b** Sleep duration—overall probability of ≥9 h sleep by years from diagnosis. Prior to diagnosis the estimated probability of long sleep increased from 3% at 20 years prior to 6% at diagnosis. The probability increased at a faster rate after diagnosis to 15% about 20 years after diagnosis (*p* = 0.43). Vertical center line indicates breast cancer diagnosis time point.

The probability of short sleep, <6 h (Fig. 2a), and long sleep, ≥9 h (Fig. 2b), were increasing prior to breast cancer diagnosis and did not significantly change following diagnosis (short sleep: β-interaction = −0.01, 95% CI: −0.04–0.01, *z* = −1.05, *p*-interaction = 0.29; long sleep: β-interaction = 0.01, 95% CI: −0.02–0.04, *z* = 0.80, *p*-interaction = 0.43). There was also no significant difference in change in trend of the probability of short or long sleep following diagnosis across race/ethnicity, age at diagnosis, cancer stage, and all other demographic variables tested (Holm’s corrected *p*-values all >0.5).

### Change in depressive symptoms

**Fig. 3:**
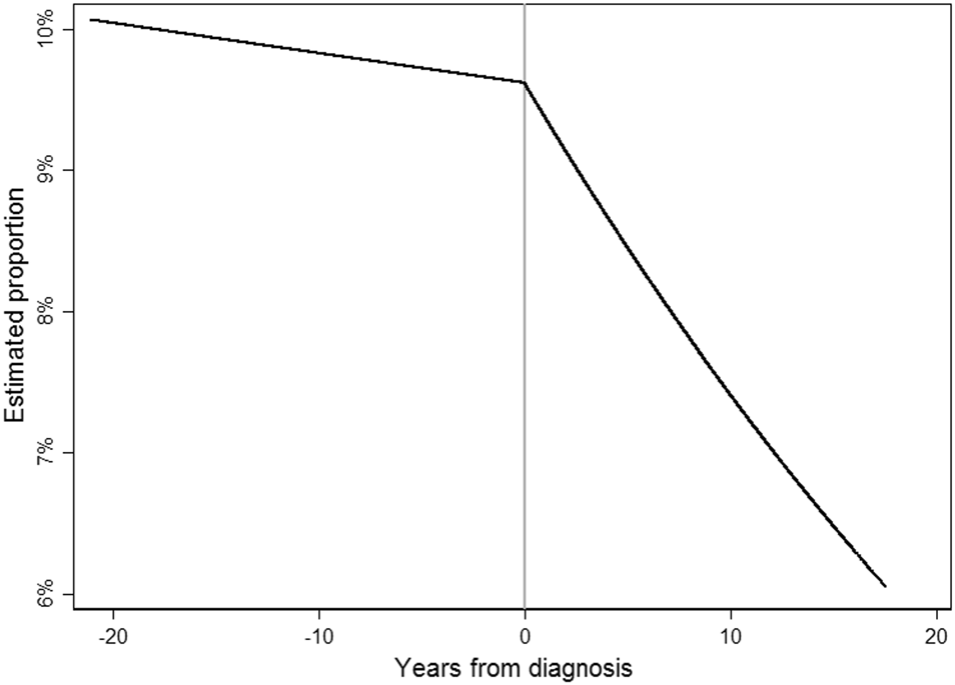
Overall probability of depression by years from diagnosis. The trend in depression levels changed following breast cancer diagnosis (*p* < 0.01). The probability of depression went from 9.5% at diagnosis to 6% about 20 years after diagnosis. Vertical center line indicates breast cancer diagnosis time point.

We investigated the trend in depressive symptoms by number of years since diagnosis and found the trend changed following breast cancer diagnosis (β-interaction = −0.04, 95% CI: −0.06–−0.01, *z* = −1.05, *p*-interaction <0.01). Prior to breast cancer diagnosis, there was a slight decreasing trend in the probability of depressive symptoms over time, but following diagnosis the probability of depressive symptoms decreased more rapidly, from 9.5% at diagnosis to only 6% about 20 years after diagnosis (Fig. 3). We also adjusted for depressive symptoms as a time-varying covariate in the WHIIRS sleep quality score model. When depressive symptoms were included, the time by period interaction was no longer statistically significant (β-interaction = −0.01, 95% CI: −0.03–0.01, *t*(6033) = −1.24, *p*-interaction = 0.22). This suggests that the changes in depressive symptoms may explain the change in the longitudinal trend in sleep quality.

### Sensitivity analysis

The results of the sensitivity analysis restricted to women with measures at both pre- and post-diagnosis time points were similar to the main results (all women, regardless of sleep measure time points), and thus the conclusions remained the same.

## Discussion

This study examined the change in longitudinal trends of sleep quality and sleep duration following breast cancer diagnosis among breast cancer survivors. Our findings demonstrated that the trend in sleep quality does significantly change following diagnosis; however, there were no statistically significant changes for sleep duration. We were unable to compare our study findings to other findings that most women’s breast cancer-related sleep problems resolve within a few years of diagnosis and treatment,^11^ given that our sleep data were on average 5.8 years after diagnosis. As a result, we may be missing immediate changes in sleep patterns following diagnosis. Due to the lengthy follow-up times post diagnosis, this paper focused on assessing long-term trends.

We found that sleep quality continues to worsen after diagnosis, but at a slower rate than before. An increase of 0.08 points per year before diagnosis to an increase of 0.06 points per year after diagnosis is a small change in sleep disturbance. While this is likely not clinically significant in 1-year increments, breast cancer survivors are now living many years after their diagnosis. Over longer time periods, increases in sleep disturbance may become more troublesome. Short sleep and other symptoms of poor sleep quality, including snoring, before diagnosis have been previously associated with breast cancer survival.^19^ These symptoms in our study continued to worsen following diagnosis, suggesting that sleep is an important health behavior to address in survivorship care.

We also hypothesized that the change in the trend of sleep quality and duration would differ when stratified by subgroups (race/ethnicity, age at diagnosis, cancer stage at diagnosis, education level, income level, BMI category, marital status, physical activity, and alcohol drinking). However, there were no statistically significant differences in the change in trend after diagnosis for each subgroup. An earlier WHI study found minimal variation in insomnia complaints due to age, race/ethnicity, exercise, cigarette, alcohol, and coffee consumption, income, employment, and marital status.^16^ A 2011 study^11^ investigated longitudinal sleep duration changes early in breast cancer survivorship and also had null findings by demographic and clinical variables. Prior studies^23–25^ demonstrated differences between sleep quality and duration by demographic variables, such as age and sociodemographic factors yet most of these studies were cross-sectional and did not examine differences in longitudinal trends, which suggests the need for more longitudinal studies to fully understand subgroup differences. The role of race/ethnicity in sleep may be very complex and could range from the biological (e.g., genetic differences, comorbidity burden) to the sociodemographic (e.g., work schedules, home environment, neighborhood deprivation).^26^ Unfortunately we did not assess all of these variables in the current study.

The probability of women having short sleep increased post-diagnosis, but the change was smaller than hypothesized based on the supporting literature.^5^ WHI sleep assessments were not collected frequently enough to observe changes in sleep which may have occurred close to diagnosis. Also, women susceptible to short sleep may already be experiencing poor sleep before diagnosis. Insomnia was present in 30% of the sample at baseline, which is much higher than the estimated 10% of the US population but consistent with some estimates in postmenopausal women.^14,15^

While counter to our initial hypothesis, the decrease in the probability of depressive symptoms is similar to results from another study.^27^ A 5-year observational study observed 48% of women were depressed in the first year after diagnosis and decreased to 15% in the fifth year of follow-up.^28^ This same study also observed patient factors, such as younger age at diagnosis and lack of social support, increased the risk of depression more than the disease or treatment-specific factors. Women in our study were older at diagnosis which could explain the lower level of depressive symptoms after diagnosis. This resulting decrease in depressive symptoms also resulted in smaller changes in sleep disturbance.

The population of older adults in the US is growing rapidly and health care systems are predicting increasing numbers of cancer diagnoses.^29^ Almost 50% of new breast cancer cases are diagnosed in women between the ages of 55 and 74 years.^1^ Older cancer survivors, often with comorbid conditions, will have complex needs and will be the focus of cancer control and prevention efforts.^30^ Breast cancer survivors currently comprise 3 million US women and 5-year survival rates are as high as 89%. Thus, it is important to know more about the projected health of aging women, including sleep.

There are some limitations to this research. The participants in this study were survivors of breast cancer, and there could be differences, such as severity of disease or age at diagnosis, between the women who did and did not survive (*n* = 2716; 22.4%) during follow-up. Therefore, the smaller effect sizes we observed may be due, in part, to survival bias. Sleep quality and duration were also measured at variable times in relation to cancer diagnosis. Some women had measures closer to their breast cancer diagnosis, whereas others were assessed later in their survivorship when they were no longer undergoing treatment and/or experiencing many other cancer-related stressors. We did not have complete data on the participants’ cancer treatment, but sleep quality changes may be related to the type, timing, and effects of cancer treatment^8,9^ so future research should include these variables when possible. If sleep patterns had been assessed more frequently in WHI, it is possible we may have captured nonlinear trends due to temporary shifts in sleep patterns immediately following breast cancer diagnosis and during treatment. Finally, sleep assessments were self-reported and we lacked an objective sleep measure, such as polysomnography or actigraphy.^31,32^ Nevertheless, self-report remains a cost effective and frequently used source of population sleep data.^33,34^

Strengths of this study are its uniqueness in reporting longer-term trends up to 20 years after diagnosis. Other researchers have highlighted the lack of longitudinal sleep data on women.^12^ Further, few research studies consider more than one aspect of sleep and we examined both self-reported sleep quality and sleep duration. Also, the large sample size and generalizability of our results, given that the WHI sample are from 40 US clinical centers representing data from many racial/ethnic minorities, are major strengths. This study focused on within-survivor differences, but future research using cancer-free controls will only further elucidate the longer term sleep impact on survivors. By examining changes in trends following diagnosis, we are able to look at whether a breast cancer diagnosis affects the underlying natural aging effect. Studying the effects of aging on longitudinal sleep patterns in healthy older women, as opposed to those with a cancer diagnosis, will be an important addition to this area of research.

In summary, our study findings suggest sleep quality and duration in postmenopausal women are likely not affected by a breast cancer diagnosis over a long period of time compared to their sleep before diagnosis. However, both sleep quality and duration do clearly continue to worsen over time. Sleep habits prior to diagnosis rather than the cancer diagnosis itself appear to dictate the longitudinal trend of sleep. The American Cancer Society/American Society of Clinical Oncology Breast Cancer Survivorship Care Guideline outlines recommendations for monitoring for adverse post-treatment symptoms, such as disturbed sleep.^35^ The guideline recommends that primary care clinicians treat sleep disturbance to help combat cancer-related fatigue. Sleep is modifiable and can be improved by behavioral changes, therapy, and pharmacotherapy when needed, which makes sleep an appealing area to target for improving patient quality of life.^36^ Our analysis adds to the understanding of complex long-term changes in sleep trends among breast cancer survivors. Based on these findings, pre-existing sleep problems will be an important variable for tailoring survivor care plans. Addressing sleep quality and duration is important in improving the health-related quality of life and general health of all breast cancer survivors.

## Methods

The WHI is a longitudinal study of 161,809 post-menopausal women that began in 1993 and recruited from 40 clinical centers across the US. Women were enrolled in either the WHI clinical trials (CT), which consisted of three overlapping components (hormone therapy, dietary modification, calcium, and vitamin D supplementation), or the OS. Details of the study designs and recruitment procedures are described elsewhere.^37^ At enrollment, women were between the ages of 50–79 years. All participants provided written informed consent and Institutional Review Boards of the participating WHI sites approved the study protocols.

The main outcome measure for this study was the Women’s Health Initiative Insomnia Rating Scale (WHIIRS).^38,39^ The WHIIRS, a measure of perceived insomnia symptoms, consists of five questions that assess insomnia and sleep quality during the past 4 weeks: “Did you have trouble falling asleep?”; “Did you wake up several times at night?”; “Did you wake up earlier than you planned to?”; “Did you have trouble getting back to sleep after you woke up too early?”; and “Overall, was your typical night’s sleep during the past 4 weeks: Very sound or restful, sound or restful, average quality, restless, or very restless?” Response categories for these items range from 0 (“No, not in the past 4 weeks”) to 4 (“Yes, 5 or more times a week”), with a summed sleep quality score of the 5 items ranging from 0 to 20. A higher WHIIRS score indicates poorer sleep quality. A WHIIRS score of ≥9 is used as a cutoff for insomnia, since women scoring at or above a 9 have scores consistent with a diagnosis of insomnia.^38,39^ In addition, four insomnia severity categories were also formed from WHIIRS scores based on score grouping by previous studies.^18,39,40^ The highest category indicates most severe and most frequent insomnia symptoms.

Sleep duration was assessed with a single item: “About how many hours of sleep did you get on a typical night during the past 4 weeks?” Response categories included ≤5 h, 6 h, 7 h, 8 h, 9 h, ≥10 h. Due to the small number of responses for 9 h and ≥10 h (4.4%), we combined these into one category of ≥9 h.

WHI CT participants were scheduled to complete the WHIIRS at baseline, year 1, and study closeout (on average, year 9). A random subsample of participants were also scheduled to complete WHIIRS at year 3, year 6, and year 9 of follow-up. The WHI OS participants were scheduled to complete the measures at baseline and study closeout. For those participants enrolled in the WHI extension studies, which began in 2005, the WHIIRS was reassessed in 2011–2012. Measurements of sleep therefore occurred at multiple time points in relation to cancer diagnosis.

Breast cancer diagnoses among WHI participants were self-reported every 6 months to 1 year during study follow-up. All reported breast cancer outcomes were confirmed by medical record and pathology report review. Final adjudication was performed at the clinical coordinating center using the National Cancer Institute’s Surveillance, Epidemiology, and End Results (SEER) coding system.

Covariates used in the analyses included age at enrollment, race/ethnicity, income, education level, marital status, body mass index (BMI) category, physical activity occurrences per week, current alcohol use, WHI study arm (OS or CT), cancer stage at diagnosis (coded using SEER^41^), sleep medications use, and depressive symptoms. Depression was assessed using the Burnam 8-item scale for depressive disorders, which includes 6 items from the Center for Epidemiologic Studies Depression Scale (CES-D) and 2 items from the Diagnostic Interview Schedule.^42^ The resulting continuous score was converted into a binary variable using a 0.06 score cutoff for depressive symptoms.^42^ For demographic variables with multiple measurements, we chose to use the closest measurement to breast cancer diagnosis without going past the diagnosis date. Age, race/ethnicity, and education were collected at baseline only. Years from enrollment in WHI to breast cancer diagnosis was calculated. Sleep measures were classified with an indicator variable to signal whether they were collected before or after breast cancer diagnosis.

The WHI offers a unique opportunity to address sleep quality and duration differences among a diverse population of aging women. Women were included in this analysis if they were: (1) diagnosed with invasive breast cancer after WHI enrollment (*n* = 12,126); and (2) had at least one completed WHIIRS and sleep duration assessment (*n* = 12, 098). Outcomes data were assessed through September 2016 and covariate data were collected through December 2015. Since we used mixed model analytic methods that allow for incomplete observations, women were included in our study sample even if they only had sleep measures before breast cancer (*n* = 4342). This strategy captures a more diverse sample of postmenopausal women diagnosed with breast cancer and greater precision of estimates compared to an analysis restricted to participants with both pre- and post-diagnosis measurements (*n* = 7706).

### Statistical analysis

Baseline demographic and clinical characteristics of the study population were calculated using means with standard deviations for continuous variables and counts with percentages for categorical variables. *χ*^2^-tests and one-way analysis of variance (ANOVA) were used to compare sleep characteristics at baseline by race/ethnicity and age group. All statistical tests were two-sided. We fit a linear mixed effects model to the longitudinal WHIIRS sleep quality data, containing slopes, which differed by period (before and after diagnosis), to examine whether the trend in WHIIRS sleep quality score over time changed following a cancer diagnosis. The time scale for the analysis was years since diagnosis. The model contained random- and fixed-effects of time since diagnosis, period, and a time-by-period interaction. The model assumed linear trends in sleep score before and after cancer diagnosis, which seemed reasonable based on LOWESS smoothed scatter plots of the data. We did not observe any differences in variability from before to after diagnosis. A Wald test of the fixed interaction effect was used to determine whether there was a change in trend following diagnosis. Additionally, we extended our model to include depression to determine if a change in depressive symptoms could explain the change in the WHIIRS sleep quality score.

To determine whether the change in trend of the WHIIRS scores post-cancer diagnosis differed by subgroup (age at diagnosis, race/ethnicity, cancer stage, education level, income level, BMI, physical activity, marital status, or alcohol use), we extended the linear mixed model. A Wald test of the three-way interaction was used to determine whether the change in trend pre- to post-diagnosis differed by subgroup. Holm’s method was used to correct for multiple three-way interaction tests for each outcome.^43^

Next, we fit a logistic regression model for sleep duration with random subject-specific intercepts accounting for the relationship between values from the same subject. Due to the categorical responses of sleep duration, we categorized sleep duration as a binary variable: As operationalized by others^33,44–46^ participants were grouped according to those who slept <6 h versus those who slept ≥6 h, to identify short sleepers and those who slept <9 h versus ≥9 h, to identify long sleepers. As in the sleep quality analysis, the sleep duration models contained fixed effects of time since diagnosis, period, and a time-by-period interaction, which was used to assess change in the trend in sleep duration following diagnosis. These models were extended to test for differences in change following breast cancer diagnosis by subgroup, using the same approach implemented in the sleep quality analysis.

Finally, we conducted a sensitivity analysis to determine if the results among women who had both pre- and post-diagnosis sleep measures (*n* = 7706) differed from the results among the entire population (*n* = 12,098), which included women with only pre-diagnosis or only post-diagnosis sleep measures (*n* = 4392).

All analyses were performed using SAS V9.4 (SAS Inc., Cary, NC) and STATA 14 (StataCorp LP, College Station, TX).

### Data availability

Data supporting the results of this paper can be requested from the Women’s Health Initiative, https://www.whi.org/researchers/SitePages/Home.aspx.

### Code availability

Additional information, including statistical code, can be requested from Chloe Beverly, Chloe.Beverly@osumc.edu.
